# Alexithymia mediates the pathway from negative life events to somatic symptoms: a cross-sectional study in a psychosomatic outpatient sample

**DOI:** 10.3389/fpsyt.2025.1680463

**Published:** 2026-01-12

**Authors:** Junjie Chen, Dandan Ma, Jinya Cao, Jing Wei

**Affiliations:** Department of Psychological Medicine, Peking Union Medical College Hospital, Chinese Academy of Medical Sciences and Peking Union Medical College, Beijing, China

**Keywords:** negative life events, alexithymia, somatic symptoms, mediating effect, outpainting

## Abstract

**Introduction:**

Somatic Symptom Disorder (SSD), characterized by distressing and functionally impairing physical symptoms without adequate medical explanation, imposes substantial personal and socioeconomic burdens. While negative life events (NLEs) and alexithymia (difficulty identifying/describing emotions) are established risk factors for somatic symptoms, their interrelationships and underlying mechanisms remain inadequately explored. This study investigated whether alexithymia mediates the pathway between NLEs and somatic symptoms in a clinical sample.

**Methods:**

A cross-sectional analysis was conducted with 523 psychosomatic outpatients (65.2% female, mean age 41.45 ± 12.36 years) from Peking Union Medical College Hospital (2020-2025). Participants completed: 1) the *Life Events Scale (LES)* to assess NLE stress burden, 2) the *Toronto Alexithymia Scale (TAS-26)* to measure alexithymia (focusing on Difficulty Identifying Feelings [DIF] and Difficulty Describing Feelings [DDF]), and 3) the *Patient Health Questionnaire-15 (PHQ-15)* to quantify somatic symptom severity. Pearson correlations, linear regression, and mediation analyses (controlling for age/gender) examined relationships and mediating effects.

**Results:**

NLEs and alexithymia were significantly correlated with somatic symptoms (r = 0.330 and r = 0.369, respectively, p<0.01). Linear regression confirmed both as significant predictors, explaining 11% and 13.6% of somatic symptom variance, respectively. Alexithymia subscales DIF (β = 0.220, p<0.001) and DDF (β = 0.141, p = 0.014) contributed significantly, while externally oriented thinking (EOT) did not. Crucially, mediation analysis revealed alexithymia partially mediated the NLEs-somatic symptoms pathway (indirect effect: B = 0.0028, 95% CI[0.001, 0.005]), accounting for 16.25% of the total effect. A bidirectional mediation effect was also observed, with NLEs mediating 11.9% of the alexithymia-somatic symptoms relationship.

**Conclusion:**

Alexithymia significantly mediates the relationship between NLEs and somatic symptoms in psychosomatic outpatients, though its effect size (16.25%) suggests additional multifactorial pathways. Conversely, NLEs also partially mediate the alexithymia-symptom relationship, indicating complex bidirectional dynamics. Clinically, interventions targeting both alexithymia and NLE exposure may alleviate somatic symptom burden.

## Introduction

Somatic symptom disorder(SSD) manifests as an excessive preoccupation with physical symptoms—such as pain, headaches, fatigue, or dyspnea—that cause significant distress and functional impairment (1). Though typically non-life-threatening, these symptoms substantially disrupt daily living, including sleep patterns, psychological well-being, and social engagement, thereby diminishing quality of life and impairing educational or occupational functioning. Furthermore, persistent somatic complaints often necessitate recurrent medical interventions, imposing considerable socioeconomic burdens (2, 3). The etiology of such medically unexplained symptoms (MUSs) remains elusive (4).

Notably, substantial evidence implicates negative life events (NLEs) as significant contributors to the pathogenesis and persistence of somatic symptomatology (5, 6). These events encompass abrupt, disruptive experiences that fundamentally alter an individual’s social environment (e.g., unemployment, discrimination, financial crisis) (7). The posited mechanisms linking adversity to somatic manifestations involve maladaptive physiological and affective stress responses (5). Such experiences typically evoke dysphoric emotional states—including anxiety, helplessness, and pervasive negativity—that catalyze somatic expressions. However, the diathesis determining whether individuals develop somatic symptoms or not in face of NLEs are still unclear so far. Therefore, it’s necessary to find out the potential mediating factors.

According to psychoanalysis theory that suppressing unconsciousness could convert to somatic symptoms, we are interested in alexithymia, a concept means ‘‘no words for feelings’’. Alexithymia is generally considered as a relatively stable personal trait that has a high comorbidity for several psychiatric disorders such as depression disorder, anxiety disorder and somatic symptoms disorder and also contributes to suicidal ideation (8–10). Alexithymia constitutes a deficit in the cognitive processing of emotions, clinically characterized by: (1) difficulties in identifying subjective feelings, (2) impairments in describing affective states, (3) an externally oriented thinking style, and (4) limited imaginative capacity (11). According to the stress-alexithymia hypothesis (12), individuals with this condition exhibit a propensity for heightened negative evaluation of environmental stimuli due to fundamental impairments in emotional recognition and description. This deficit concomitantly reduces stress-coping efficacy. Such inaccurate evaluation of environmental challenges and threats ultimately engenders persistent stress states in individuals with alexithymia (13). Finally, intense stress may lead to an increased autonomic nervous system activity and heightened neuro-endocrine responses, creating conditions that may be conducive to the development of somatic disorders (14). Previous study has proved that alexithymia is positively correlated to somatization or somatic symptoms with a small to moderate effect (6, 15). Compared to healthy control, SSD patients have a higher degree of alexithymia (16, 17). These results infer that alexithymia plays an important role in somatic symptoms pathogenesis.

While the correlations between these variables appear well-established individually, the underlying mechanisms connecting NLEs, alexithymia, and somatic symptoms remain insufficiently explored.

Previous neuroimaging research on functional connectivity (FC) in individuals with somatic symptom disorder (SSD) has demonstrated not only elevated levels of somatosensory amplification compared to healthy controls but also a correlation between this amplification and increased FC linking the somatosensory network (SMN) with the salience and dorsal attention networks (18). These findings are further supported by studies indicating that the SMN in SSD exhibits strong functional connections with the default mode network (DMN), the salience network, and other networks involved in affective processing which is highly related to alexithymia (19, 20). This study aims to investigate the interrelationships among negative life events (NLEs), alexithymia, and somatic symptoms through the following objectives: (1) to elucidate the complex associations between NLEs, alexithymia, and somatic symptoms; and (2) to develop a mediating effect model elucidating these relationships using clinical samples from Chinese outpatient settings.

## Method and material

### Participants

Patients who visited the outpatient clinic of the Department of Psychological Medicine at Peking Union Medical College Hospital between June 2020 and March 2025 and completed the specified set of questionnaires were included in the study. The primary diagnoses in our clinic encompass anxiety disorders, depressive disorders, somatic symptom disorders, insomnia, and related conditions. For patients with multiple visits, only the questionnaire results from their initial visit were considered. The study protocol received approval from the Ethics Committees of Peking Union Medical College Hospital, and the research was conducted in compliance with the latest version of the Declaration of Helsinki. Written informed consent was obtained from all participants prior to their involvement in the study.

### Measurements

#### Negative life events

The Life Events Scale (LES), a self-administered questionnaire comprising 48 items, was applied to assess negative life events (NLEs). This scale, validated and widely applied in Chinese populations (21, 22), evaluates the perceived stressfulness and frequency of stressful life events across three domains: family life (28 items), work and study (13 items), and social and other aspects (7 items). It encompasses NLEs such as bereavement, severe illness, unemployment, financial difficulties, and social challenges. Each event is assessed based on five criteria: event type, timing, impact severity, duration of impact, and frequency of occurrence. The perceived stress score is calculated by multiplying the severity, duration, and frequency scores. Higher LES scores reflect greater perceived stress levels (23). In this study, the LES demonstrated good internal consistency, with a Cronbach’s alpha of 0.82.

#### Somatic symptoms

Somatic symptoms were measured by the Patient Health Questionnaire-15 (PHQ-15) (24). The PHQ-15 is a reliable and valid self-report questionnaire designed to assess the severity of somatic symptoms. The scale comprises 15 items, each scored from 0 (not bothered at all) to 2 (bothered a lot), resulting in a total score ranging from 0 to 30. The Chinese version of the PHQ-15 has demonstrated robust reliability and validity in previous studies. In this study, the PHQ-15 demonstrated good internal consistency, with a Cronbach’s alpha of 0.81.

#### Alexithymia

Alexithymia was evaluated using the Toronto Alexithymia Scale (TAS-26), a 26-item self-report measure with established reliability and validity across diverse populations (25). This 5-point Likert scale ranges from 1 (strongly disagree) to 5 (strongly agree), yielding a total score between 26 and 130, where higher scores indicate greater alexithymia severity. The TAS-26 comprises four subscales, three of which were included in our analysis: (1) difficulty describing feelings (DDF), (2) difficulty identifying feelings (DIF), and (3) externally oriented thinking (EOT). The fourth subscale (limited daydreaming) was excluded, as Taylor et al. (26) found it lacked correlation with the other factors. Consequently, the total score in this study was derived from the sum of the first three subscales. The TAS-26 demonstrated strong internal consistency in our sample, with a Cronbach’s alpha of 0.718.

### Data analysis

Continuous variables are expressed as mean ± standard deviation (SD), while categorical variables are reported as percentages. The Pearson correlation coefficient was employed to examine the relationships among negative life events (NLEs), alexithymia, and somatic symptoms. To further explore these associations, linear regression analyses were conducted with NLEs and alexithymia as predictor variables and somatic symptom severity as the dependent variable, adjusting for participants’ gender and age. Stepwise regression was utilized to identify subscales significantly associated with somatic symptoms and to derive a mathematical model representing the relationship between somatic symptoms, NLEs, and alexithymia. All statistical analyses were performed using IBM SPSS Statistics 26.0, with a two-tailed significance level set at *p* < 0.05.

Using the Bootstrap method with 5,000 samples, the mediating role of alexithymia in relationship between NLEs and somatic symptoms was tested through SPSS PROCESS (Model 4) (27). Gender (code: male = 1, female = 2) and age were included as covariates to control for potential demographic impacts. The reason selecting gender and age as covariate is these two factors have significant influence on most psychiatric disorder (28, 29).

## Results

### Participant characteristics

In this study, a total of 523 outpatients were recruited, among them, 341 (65.20%) were females and 182 (34.80%) were males. The age of participants ranged from 18 to 79 years (M = 41.45, SD = 12.36). The total score of PHQ-15 ranged from 1 to 30 with a mean of 13.79 (SD = 6.02). Among them, 380 (72.66%) had a PHQ-15 score of ten or higher, which means clinically significant symptom. The mean score of the NLEs was 92.19 (SD = 110.33). The mean score of the TAS was 57.79 (SD = 10.7) (**Table 1**).

**Table 1:** Participant characteristics.

Characteristics	Total outpatients(n=523)
Gender(female, %)	65.20%
Age(M ± SD)	41.45 ± 12.36
PHQ-15 total score(M ± SD)	13.79 ± 6.02
No symptoms (0–4, %)	5.16
Mild symptoms (5–9, %)	22.18
Moderate symptoms (10–14, %)	30.4
Moderately-severe symptoms (15–19, %)	23.14
Severe symptoms (20–30, %)	19.12
LES-Negative Life Events total score (M ± SD)	92.19 ± 110.33
TAS-Difficulty in Describing Feelings score (M ± SD)	16.43 ± 5.37
TAS-Difficulty in Identifying Feelings score (M ± SD)	22.04 ± 5.09
TAS-Externally Oriented Thinking score (M ± SD)	19.32 ± 3.58
TAS total score (M ± SD)	57.79 ± 10.7

### Correlational findings

#### Correlation between clinical variables

Pearson correlation analysis showed that PHQ-15 total score, TAS total score and NLEs were significantly correlated with each other (**Table 2**).

**Table 2:** Correlations between negative life events, alexithymia and somatic symptoms.

Variables	1	2	3
1. Negative Life Events	1		
2. Alexithymia	0.163^**^	1	
3. Somatic Symptoms	0.330^**^	0.369^**^	1

^**^p < 0.01.

PHQ-15 total score was significantly and positively correlated with NLEs, positive life events (PLEs), difficulty in describing feelings and difficulty in identifying feelings (p < 0.01), however, it wasn’t correlated with externally oriented thinking (**Supplementary Table 1** and **Supplementary Table 2**). In addition, three dimension of LEs including family, work and social were also positively correlated with PHQ-15 total score (**Supplementary Table 2**).

#### Linear regression analyses

The analysis revealed that negative life events (NLEs) and alexithymia were significant predictors of somatic symptoms after accounting for the effects of age and gender (p < 0.01). Notably, NLEs accounted for 11% of the variance in somatic symptoms, while alexithymia explained 14% of the variance in somatic symptoms (**Table 3**).

**Table 3:** Linear regression model with negative life events, alexithymia as predictor variables predicting somatic symptoms.

Dependent variables	Predictors	B	β	p	t	R²	F
somatic symptoms	Negative Life Events	0.018	0.330	< 0.001	7.971	0.109	63.541
	Alexithymia	0.207	0.369	< 0.001	9.050	0.136	81.906

#### Stepwise regression analyses

The EOT subscale was not statistically significant and was consequently excluded during the stepwise regression modeling process. Conversely, negative life events (NLEs), difficulty describing feelings (DDF), and difficulty identifying feelings (DIF) all demonstrated significant positive correlations with somatic symptoms (p < 0.001) (**Table 4**). A linear regression model for somatic symptoms was established as follows:[PHQ-15] = 4.193 + 0.014 × [LES-NLEs] + 0.158 × [TAS-DDF] + 0.260 × [TAS-DIF] (p < 0.05).

**Table 4:** Stepwise regression results for somatic symptoms and variables.

Predictors	B	β	p	t	VIF	R2	F
Negative Life Events	0.014	0.252	< 0.001	6.318	1.057	0.217	47.887
TAS-1	0.158	0.141	0.0145	2.451	2.198		
TAS-2	0.260	0.220	< 0.001	3.786	2.236		

B: unstandardized beta regression coefficient; β: standardized beta regression coefficient; TAS-1: TAS-Difficulty in Describing Feelings; TAS-2: TAS-Difficulty in Identifying Feelings.

The model’s R² value (coefficient of determination) was 0.217, which indicates that NLEs and the facets of alexithymia (specifically DDF and DIF) together explained 21.7% of the observed variance in somatic symptoms among the study participants.

### Mediation results

**Figure 1:**
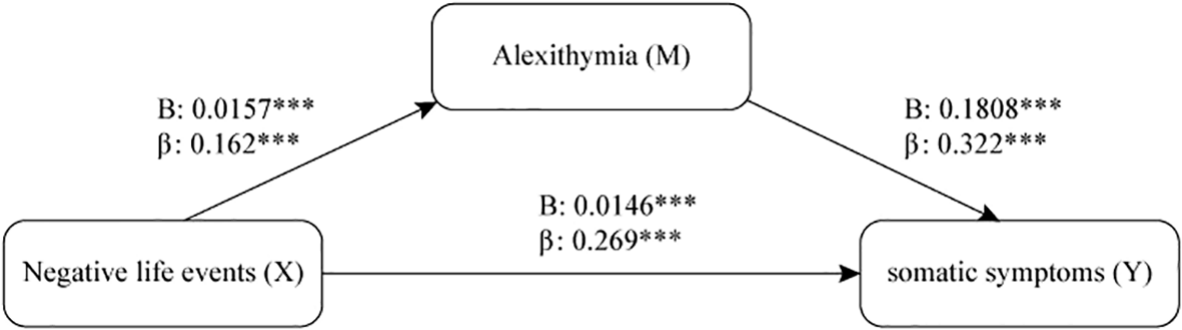
A mediating model with direct and indirect effects between negative life events, alexithymia, and somatic symptoms. The effects of gender and age were controlled in the model. β: Standardized beta regression coefficient; (B): Unstandardized beta regression coefficient. *** p < 0.001.

Alexithymia mediated the relationship between negative life events and somatic symptoms, with significant indirect effects observed (B = 0.0028, p < 0.001). While a direct NLEs-somatic symptoms pathway was also confirmed (B = 0.0146, p < 0.001), the mediating effect (a*b) of alexithymia accounted for 16.25% of the total effect from NLE to somatic symptoms (c) (**Figure 1** and **Table 5**). When applying alexithymia as independent factor and NLE as mediating factor, NLE also showed significant mediating effect in the pathway from alexithymia to somatic symptoms (**Supplementary Table 3**), whose proportion of the mediation effect (a*b/c) is approximately 11.9%.

**Table 5:** Mediating effect in mediation model.

Effect	B (95% CI)	SE	β	p
X→M(a)	0.0157 (0.007, 0.025)	0.0042	0.162	< 0.001
M→Y (b)	0.1808 (0.138, 0.222)	0.0220	0.322	< 0.001
Total effect: X→Y (c)	0.0175 (0.013, 0.022)	0.0022	0.321	< 0.001
Direct effect: X→Y (c’)	0.0146 (0.010, 0.019)	0.0021	0.269	< 0.001
Indirect effect: X→M→Y (a*b)	0.0028 (0.001, 0.005)	0.0008	0.052	< 0.001

X: NLEs, M: alexithymia; Y: somatic symptom; B: unstandardized beta regression coefficient; β: standardized beta regression coefficient; S.E.: Standard Error; 95% CI: 95% confidence interval.

## Discussion

The Patient Health Questionnaire-15 (PHQ-15), validated in prior studies (24, 30) for assessing somatic symptoms, was employed in this study to evaluate patients’ somatic symptom burden. A total of 523 patients were enrolled, with females comprising 65.2% of the cohort. Since the PHQ-15 assesses somatic symptoms without differentiating between organic and non-organic etiologies, it was not utilized for diagnostic categorization in this research.

Results indicated that over 70% of patients scored ≥10 on the PHQ-15, suggesting the presence of moderate-to-severe somatic symptoms. This finding underscores the increasing prominence of somatic symptom challenges in China, highlighting an urgent need for clinical attention and systemic solutions.

### LEs and somatic symptoms

Our study demonstrated that both positive and negative life events (PLEs and NLEs) are significantly associated with somatic symptoms. And we found all three dimensions of LEs (family, work and social) were related to somatic symptoms additionally. Notably, NLE emerged as a robust predictive factor, accounting for approximately 11% of the variance in somatic symptoms. The observed correlation between PLEs and somatic symptoms may stem from the nature of PLEs itself as a potential stress source (31), thereby contributing to the manifestation of physical symptoms.

Extensive prior research has established that life events substantially increase the risk of somatic disorders, including diabetes mellitus, hypertension, and coronary heart disease (CHD) (32–34). Specifically, patients experiencing acute coronary events report significantly elevated stress levels (35). Concurrently, individuals with pre-existing somatic disorders or persistent somatic symptoms are more susceptible to psychological distress (36), which subsequently acts as an initiating factor in a vicious cycle of negative stress.

### Alexithymia and somatic symptoms

Our findings indicate that alexithymia accounted for 13.6% of the variance in somatic symptoms and positively correlated with PHQ-15 total score, consistent with prior evidence establishing its role in somatic symptom development (15, 37, 38).

Crucially, differential effects emerged across alexithymia subcomponents: both Difficulty Describing Feelings (DDF) and Difficulty Identifying Feelings (DIF) demonstrated robust correlations with PHQ-15 scores (p<0.01), whereas Externally Oriented Thinking (EOT) showed no significant association in Pearson correlation and stepwise regression analyses. This finding was consistent with previous research (15). External-oriented thinking (EOT) has been reported to be associated with the visualization and processing of negative images (39), which is related to emotion regulation strategy, with high levels of EOT tending to interpret emotional responses as somatic symptoms (40). However, EOT-related emotion regulation strategies, compared to expressive suppression partially ​represented by DIF and DDF, appear to represent a more adaptive approach by decreasing the intensity of negative emotion responses and so automatic responses, which are easily misinterpreted as somatic symptoms (41). Therefore, EOT didn’t demonstrate a significant association with somatic symptoms.

### The mediate effect of alexithymia

A lot of study has proved significant mediating effect of alexithymia between different psychopathological, psychological or behavior traits. Huo et al. found that alexithymia played a mediating role in the pathway from cognitive deficits to negative symptoms (42). Besides, a study by Li et al. enrolling 262 adolescents with depression proved the mediating effect of alexithymia on the relationship between physical neglect and suicidal ideation (43). A study including 2747 outpatients aged from 18 to 65 by Xie et al. found alexithymia had significant mediating effect between depressive symptoms and NLEs (44).

As mentioned above, the strong association between life events and somatic symptoms has been well-documented in previous studies, and extensive research has confirmed that alexithymia increases the risk and severity of somatic symptoms. However, the mediating factor in how life events influence somatic symptoms has not been fully elucidated. Classic psychoanalytic theory posits that repressed unconscious conflicts can be converted into somatic symptoms. Given that alexithymia involves difficulties in identifying and expressing emotions, we hypothesized that when individuals experience stressful life events, alexithymia mediates the development of somatization symptoms. Our study found that alexithymia played a significant mediating role in the relationship between NLEs and somatic symptoms, accounting for 16.25% of the total effect.

Interestingly, we also found NLEs showed similar and significant proportion of mediating effect (11.9%) in the pathways from alexithymia to somatic symptoms. Possible theory explaining this phenomenon is that individuals with alexithymia have difficulty processing emotions, which makes them more vulnerable to the impact of negative life events (45). These events then trigger maladaptive psychological processes, such as increased rumination or decreased self-esteem, that exacerbate emotional distress (46, 47). Since the distress cannot be expressed emotionally, it is manifested physically through somatic symptoms, completing the mediating pathway from alexithymia to somatic symptom disorder (45).

Related to somatic symptoms, a study conducted by Xu et al. found that alleviation of alexithymia played a mediating role in the relief of psychosomatic distress in SSD patients after a mindfulness-based cognitive therapy program with a mediating proportion of 38.4% (48). The high mediating ratio may be due to cognitive behavior therapy majorly improved patient psychological function like emotion identification and description. Besides, loneliness seemed to have a larger effect on somatic symptoms with R^2^ of 0.55 in the study of Hutten et al (49).

A systematic review and meta-analysis (50) showed a variety of biological, social and psychological factors had strong correlation to persistent somatic symptoms which cannot be fully attributed to objectively determined anatomical or functional disease severity (51, 52). There factors included infection, abdominal pain, sleep problems, anxiety, depression, life events, childhood adversity, employment, health care utilization and so on. Therefore, it’s reasonable to infer multiple factors contribute to somatic symptoms development regardless of with or without biological pathology, which explains why the proportion of mediating effect of alexithymia between NLE and somatic symptoms is not so high to some extent. This further refutes the psychoanalytic view that alexithymia plays a central role in the development of somatic symptom disorders, demonstrating instead a biopsychosocial multifactorial promotion mechanism (53).

## Limitation

The current study has several limitations. First, the reliance on self-reported questionnaires for assessing NLEs, alexithymia, and somatic symptoms may introduce recall bias and common method variance. Furthermore, confounding factors such as participants’ educational background and comprehension abilities could have influenced response accuracy. Future studies would benefit from employing multimethod assessment strategies, such as supplementing self-reports with clinician-rated instruments (e.g., standardized diagnostic interviews for SSD) and objective physiological markers of stress (e.g., cortisol levels) to triangulate findings and improve validity. Second, although we controlled for age and gender, a more comprehensive set of baseline assessments incorporating key sociodemographic variables (e.g., socioeconomic status, ethnicity) and biological factors (e.g., smoking, substance abuse, family history) would allow for a more thorough evaluation of potential confounders and enhance the robustness of the findings. Third, the cross-sectional design of this investigation only allows for the assessment of correlation patterns, thereby limiting our ability to establish causal relationships among NLEs, alexithymia, and somatic symptoms. To address this, future longitudinal studies​ with multiple time points are necessary to clarify the temporal dynamics and directional pathways between these variables. Such designs could also explore whether early psychological interventions targeting alexithymia can effectively mitigate the development or worsening of somatic symptoms over time. Finally, as our study population was not categorized based on a clinical diagnosis of somatic symptom disorder (SSD), the persuasiveness of our conclusion for direct clinical practice is somewhat limited. To significantly increase the clinical relevance and translational impact of future research, it is essential to conduct stratified analyses in well-defined clinical cohorts​ meeting diagnostic criteria for SSD. This would help determine whether the role of alexithymia is generalizable across different levels of symptom severity and specific diagnostic categories.

## Conclusion

Negative life events (particularly NLEs) and alexithymia are significant risk factors for somatic symptoms. Alexithymia demonstrates a substantial mediating effect between NLEs and somatic symptoms. Notably, NLEs conversely exhibit a significant mediating effect between alexithymia and somatic symptoms. Psychological intervention like cognitive behavior therapy reducing negative life events’ impact and improving patients’ alexithymia could help alleviate somatic symptoms (48, 54). Future exploratory investigations should validate the causal relationships among these three factors and develop targeted interventions.

## Data Availability

The raw data supporting the conclusions of this article will be made available by the authors, without undue reservation.
