# Supplementary material for: Alexithymia mediates the pathway from negative life events to somatic symptoms: a cross-sectional study in a psychosomatic outpatient sample
Source: Front Psychiatry. 2026 Jan 12;16:1680463. doi: 10.3389/fpsyt.2025.1680463 (PMC12832783; doi:10.3389/fpsyt.2025.1680463)
Supplement: Supplementary file 1 [file Table1.docx]

**Supplementary Table 1**

**Correlations among study variables (n=523)**

| Variables | 1 | 2 | 3 | 4 | 5 |
| --- | --- | --- | --- | --- | --- |
| 1. Negative Life Events | 1 |  |  |  |  |
| 2. Difficulty in Describing Feelings | 0.191^**^ | 1 |  |  |  |
| 3. Difficulty in Identifying Feelings | 0.230^**^ | 0.738^**^ | 1 |  |  |
| 4. Externally Oriented Thinking | -0.126^**^ | 0.132^**^ | 0.038 | 1 |  |
| 5. Somatic Symptoms | 0.330^**^ | 0. 352^**^ | 0.382^**^ | 0.031 | 1 |

^**^*p* < 0.01.

**Supplementary Table 2**

**Correlations among life events and PHQ-15 total score (n=523)**

| Variables | PHQ-15 total score | P value |
| --- | --- | --- |
| 1. Positive life events | 0.1151 | 0.008 |
| 2. Negative life events | 0.3297 | ＜0.001 |
| 3. Family | 0.2471 | ＜0.001 |
| 4. Work | 0.2882 | ＜0.001 |
| 5. Social | 0.2283 | ＜0.001 |

**Supplementary Table 3**

**NLE Mediating effect in mediation pathways from alexithymia to somatic symptoms (n=523)**

| **Effect** | **B (95% CI)** | **SE** | **β** | **p** |
| --- | --- | --- | --- | --- |
| X→M(a) | 1.672 (0.788, 2.605) | 0.447 | 0.162 | < 0.001 |
| M→Y (b) | 0.0146 (0.010, 0.019) | 0.002 | 0.269 | < 0.001 |
| Total effect: X→Y (c) | 0.2053 (0.162, 0.247) | 0.023 | 0.365 | < 0.001 |
| Direct effect: X→Y (c') | 0.1808 (0.138, 0.222) | 0.022 | 0.322 | < 0.001 |
| Indirect effect: X→M→Y (a*b) | 0.0245 (0.010, 0.042) | 0.007 | 0.044 | < 0.001 |

X: alexithymia; M: NLE; Y: somatic symptom; B: unstandardized beta regression coefficient; β: standardized beta regression coefficient; S.E.: Standard Error; 95% CI: 95% confidence interval.
